# 5-HT1a activation in PO/AH area induces therapeutic hypothermia in a rat model of intracerebral hemorrhage

**DOI:** 10.18632/oncotarget.20280

**Published:** 2017-08-16

**Authors:** Tan Liang, Qianwei Chen, Qiang Li, Rongwei Li, Jun Tang, Rong Hu, Jun Zhong, Hongfei Ge, Xin Liu, Feng Hua

**Affiliations:** ^1^ Department of Neurosurgery, Southwest Hospital, Third Military Medical University, Chongqing, China

**Keywords:** intracerebral hemorrhage, therapeutic hypothermia, 8-OH-DPAT, 5-HT1a, PO/AH area

## Abstract

Therapeutic hypothermia is widely applied as a neuroprotective measure on intracerebral hemorrhage (ICH). However, several clinical trials regarding physical hypothermia encountered successive failures because of its side-effects in recent years. Increasing evidences indicate that chemical hypothermia that targets hypothalamic 5-HT1a has potential to down-regulate temperature set point without major side-effects. Thus, this study examined the efficacy and safety of 5-HT1a stimulation in PO/AH area for treating ICH rats. First, the relationship between head temperature and clinical outcomes was investigated in ICH patients and rat models, respectively. Second, the expression and distribution of 5-HT1a receptor in PO/AH area was explored by using whole-cell patch and confocal microscopy. In the meantime, the whole-cell patch was subsequently applied to investigate the involvement of 5-HT1a receptors in temperature regulation. Third, we compared the efficacy between traditional PH and 5-HT1a activation-induced hypothermia for ICH rats. Our data showed that more severe perihematomal edema (PHE) and neurological deficits was associated with increased head temperature following ICH. 5-HT1a receptor was located on warm-sensitive neurons in PO/AH area and 8-OH-DPAT (5-HT1a receptor agonist) significantly enhanced the firing rate of warm-sensitive neurons. 8-OH-DPAT treatment provided a steadier reduction in brain temperature without a withdrawal rebound, which also exhibited a superior neuroprotective effect on ICH-induced neurological dysfunction, white matter injury and BBB damage compared with physical hypothermia. These findings suggest that chemical hypothermia targeting 5-HT1a receptor in PO/AH area could act as a novel therapeutic manner against ICH, which may provide a breakthrough for therapeutic hypothermia.

## INTRODUCTION

Spontaneous intracerebral hemorrhage (ICH), as a subtype of stroke, accounts for approximately two million (10-15%) of 15 million stroke patients worldwide each year, and leads to a higher rates of death and disability than ischemic stroke [1]. White matter injury, neuronal necrosis and blood brain battier (BBB) damage during the acute stage of ICH contribute to the early brain injury development, which is associated with a majority of deaths in the early stage [2]. Therapeutic hypothermia has been widely used in treating the patients with acute traumatic brain injury [3], stroke [4] and neonatal hypoxic-ischemic encephalopathy [5]. Accumulated clinical evidences indicated that therapeutic hypothermia could protect ICH patients against early brain injury [6, 7].

Therapeutic hypothermia could be achieved *via* surface, intravascular or pharmacological routes, among which physical cooling was the most extensively used one in clinical practices [8]. Unfortunately, most of previous multi-center randomized clinical trials failed to verify the neuroprotective effect of physical hypothermia (PH) for ICH [9-11]. These successive failures are likely due to the side-effects of exogenous cooling methods [12]. The vigorous thermo-regulatory defense including shivering would be triggered by the exogenous cooling process and elicits a systemic stress reaction, which could lead to insufficient blood supply to the lesion area [13]. Furthermore, the rapid increase in intracranial pressure (ICP) during the rewarming process may also contribute to the failure of PH for ICH [11]. There has been a recent surge of interest in the investigation of pharmacological hypothermia as a treatment option for central nervous system (CNS) injuries. Several classes of neuroprotective agents have been shown to be involved in central thermoregulation. TAK-937, a Cannabinoid 1 receptor agonist, has been reported to have hypothermic effects by achieving 34.1 ± 0.7 °C at 5 hour after administration. Its neuroprotection role has been demonstrated on a rat model of transient middle cerebral artery occlusion [14]. Recently, the novel neurotensin receptor 1 agonists such as HPI 201 and HP I363 were reported to reduce 3-5 °C in body temperature of rats and showed promising results in different models of CNS injury [15-17]. Thus, it is needed to explore more pharmacological-induced hypothermia avenue to overcome the “bottle neck” in therapeutic hypothermia for ICH.

As reported, the increased extracellular concentration of 5-HT in the hypothalamus was associated with down-regulation of the temperature set point [18]. The neuroprotective effects of selective serotonin reuptake inhibitors (SSRIs) against ischemic stroke have been attested by clinical and animal experiments; however, the underlying mechanism remains unclear [19, 20]. Presumably, chemical hypothermia (CH) that targets hypothalamic 5-HT system could directly down-regulates the temperature set point without causing shivering [21]. Moreover, compared with the surface or intravascular cooling routes, the way of CH therapy was reported to be much gentler in cooling induction and rewarming [22]. Therefore, hypothalamic 5-HT might be a new therapeutic target for therapeutic hypothermia for ICH.

8-hydroxy-2-(di-n-propylamino) tetralin (8-OH-DPAT), as a potent 5-HT1a receptor agonist, has been demonstrated with neuroprotective effects against L-DOPA-induced dyskinesia [23], brain trauma [24] and global cerebral ischemia [25]. With direct activation of endogenous 5-HT1a receptor, 8-OH-DPAT also exhibits a similar anti-depression effect like SSRIs and a stronger effect on the thermotaxic center [26]. Based on this, we subsequently compared the neuroprotection of 8-OH-DPAT with physical cooling on ICH animals in present study. The mechanism of chemical hypothermia achieved by 8-OH-DPAT was further investigated *via* a functional assessment of the warm-sensitive neurons in preoptic anterior hypothalamus (PO/AH) area. It is proposed that these findings are of relevance to a potential, novel hypothermia therapy to ameliorate the early brain injury of ICH patients without major side-effects.

## RESULTS

### Early increased brain temperature was related to worse outcomes in ICH patients

**Figure 1:**
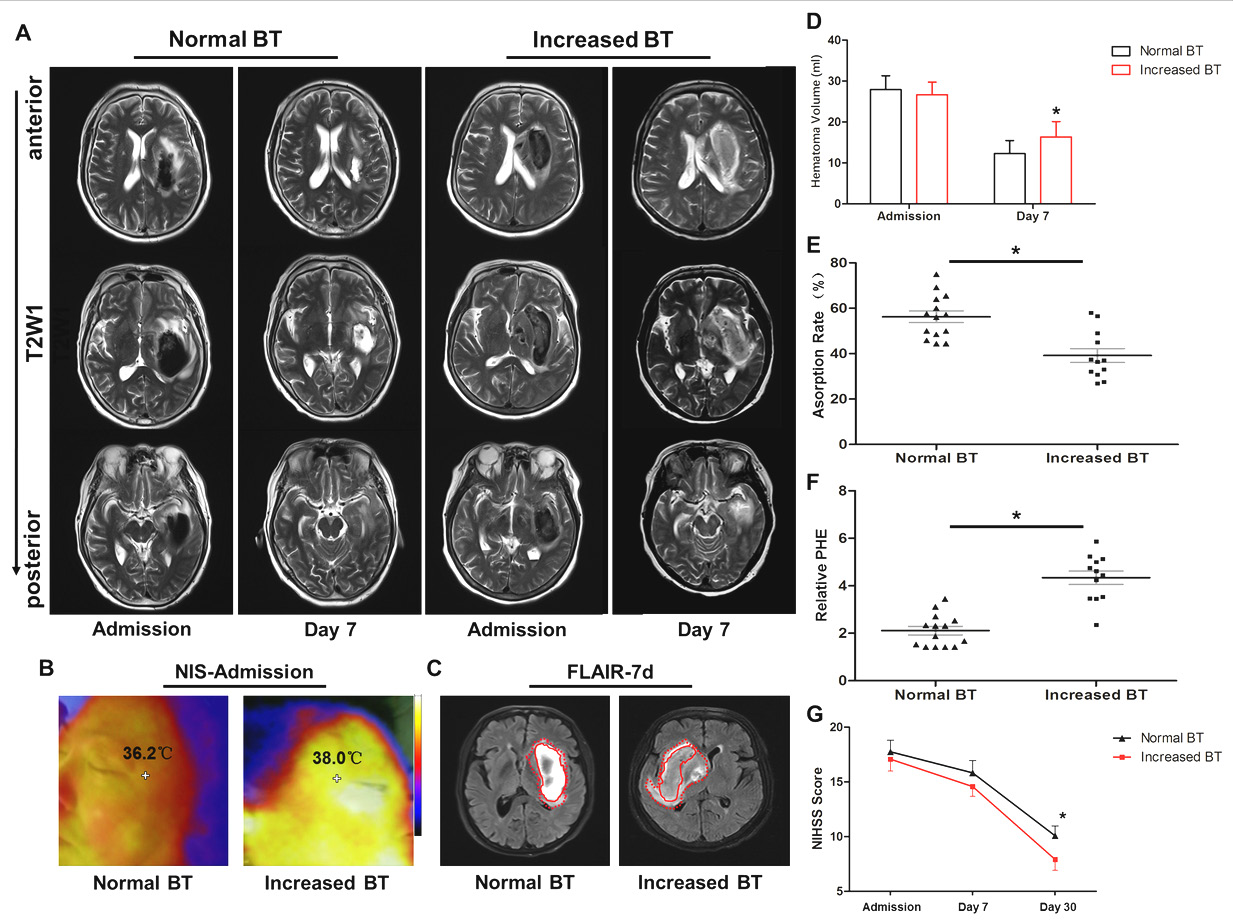
Increased brain temperature reduced hematoma absorption and aggravated neurological deficits in intracerebral hemorrhage (ICH) patients **A.** Typical cranial MRI-T2 scans (coronal brain section) of normal BT (*n* = 14) and increased BT (*n* = 12) subjects at admission and day 7 after onset. **B.** Near-infrared spectrum (NIS) images of ICH patients in the normal BT and increased BT groups at admission. The temperature measuring point was set at the ipsilateral tempus. **C.** Margins of hematoma are outlined by the red full curve, and peri-hematomal edema is outlined by the red dotted line in the FLAIR images of the normal BT and increased BT subjects at 7 days following ICH. **D.** Evolution of the hematoma volume was quantified by a histogram at admission and 7 days after the onset of ICH. **E.** Comparison of the hematoma absorption rate indicated a decreased tendency in the increased BT group at 7 days after ICH. **F.** Significant decrease in the relative peri-hematoma edema was identified in the increased BT group compared with the normal BT group at 7 days after ICH. **G.** There was a reduced NIHSS score in the increased BT group at 30 days after ICH. * *p* < 0.05 increased BT *vs*. normal BT group. Values represent means ± SDs. Paired *t*-test.

There was no significant difference between the hematoma volumes of normal and increased BT patients on admission; however, a significant higher hematoma absorption rate was identified in normal BT patients than increased BT patients at 7 d after onset (Figure 1A and 1E). As shown in Figure 1C, the edema volumes of ICH patient rapidly expanded in the first 7 d of onset. The relative PHE volume of increased BT patients was substantially larger than the patients in normal BT group at 7 d (Figure 1F). Moreover, the NIHSS scores of increased BT patients at 30 d were significantly lower than normal BT group (Figure 1G). As shown in Table 1, the patients of two groups shared similar clinical baseline.

**Table 1:** Baseline characteristics of enrolled ICH patients

	Normal BT (*n*=14)	Increased BT (*n*=12)	*P*-Value
Age, y	61.4	58.7	NS
Sex (M/F, %)	55.5	62.5	NS
onset to admission, h	9.4 (5-17)	8.9 (4-15)	NS
Medical history, (%)			
Hypertension	78.6	75.0	NS
Diabetes	28.6	25.0	NS
lipid metabolism disorder	21.4	16.7	NS
Smoking habit (current)	28.6	33.3	NS
Medication, (%)			
Warfarin anticoagulation	0	0	NS
Anti-platelet drugs	28.6	33.3	NS
Ant-diabetic drugs	21.4	25.0	NS
Clinical features			
Blood pressure, mm Hg			
Systolic	172 (45)	169 (42)	NS
Diastolic	105 (12)	102 (14)	NS
Body temperature, ( C°)	36.6 (0.5)	37.4 (0.5)	NS
Glasgow scale	13.1 (1.5)	14.1 (1.7)	NS
NIHSS	17.8 (1.1)	17.1 (1.1)	NS
ICH volume	27.9 (3.4)	26.7 (3.1)	NS
Serum glucose, mg/dL	124 (22)	129 (31)	NS
Platelet count, ×10^3^/mmc	189 (62)	174 (53)	NS
Leukocyte count, ×10^3^/mmc	7.6 (1.1)	7.9 (0.9)	NS

Values are presented as proportions, mean (SD), or median (quartiles). NIHSS: National Institute of Health Stroke Scale; ICH: intracerebral hemorrhage; mmc: cubic millimeter.

### 8-OH-DPAT alleviated the increased brain temperature induced by ICH

**Figure 2:**
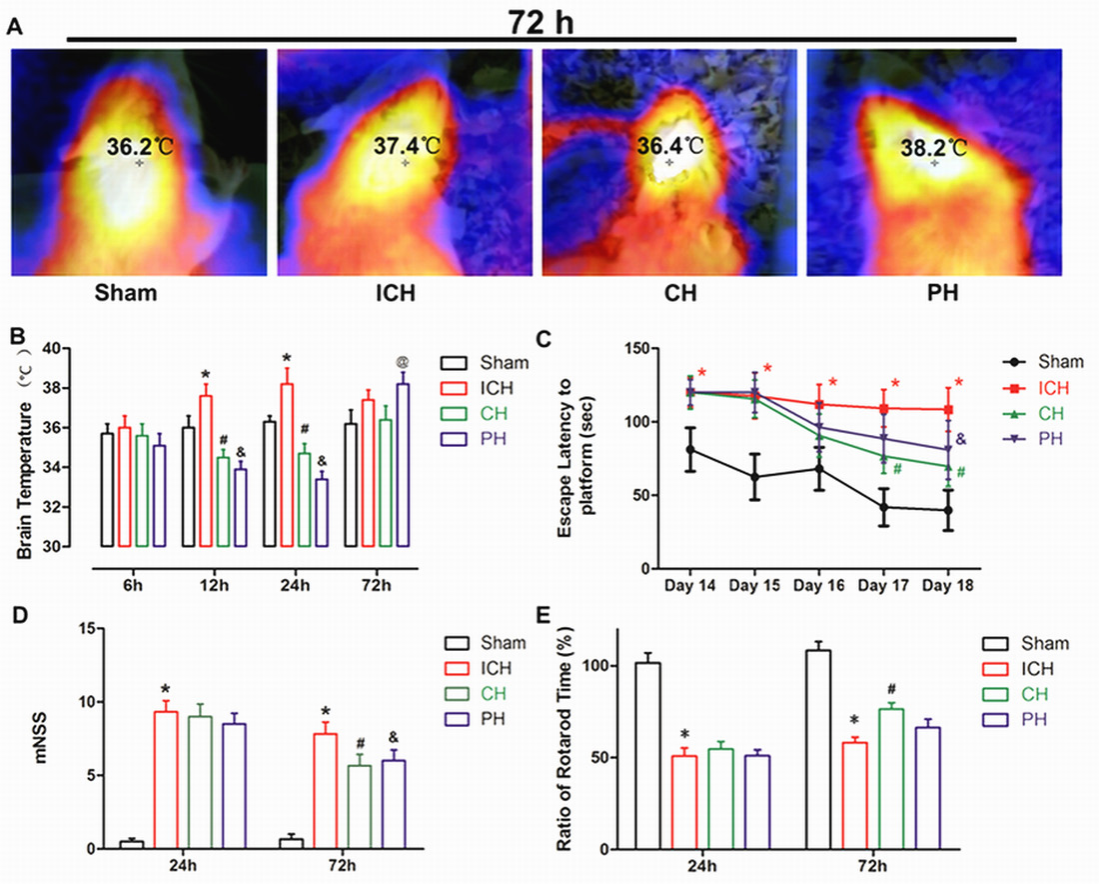
Early increased brain temperature was associated with worse neurologic outcomes in intracerebral hemorrhage (ICH) rats **A.** Representative thermal images of the experimental animals in the four groups at 72 h after surgery. **B.** Brain temperature measured by surface near-infrared spectrum imaging at 6, 12, 24 and 72 h after infusion. **C.** Escape latency was recorded in the Morris water maze at days 14-18 after ICH. **D.** mNSS score in the four groups was presented by a histogram at 24 and 72 h after ICH. **E.** Ratio of the rotarod time was recorded at 24 and 72 h after the onset of ICH. * *p* < 0.05 ICH group *vs*. Sham group. # *p* < 0.05 CH group *vs*. ICH group.&*p* < 0.05 PH group *vs*. ICH group. @ *p* < 0.05 PH group *vs*. ICH group. The bar represents the mean ± SD. One-way ANOVA and Student-Newman-Keuls analysis. *n* = 9 in the sham group and *n* = 8 in the remaining groups at each time point.

For the rat model of ICH, the brain temperature was determined with a Fluke VT04 thermometer at the corresponding time points after ICH (Figure 2A). The increased temperature of ICH rats reached a peak at 24 h and then gradually decreased to a relatively normal level at 72 h following ICH. For PH-treated animals, the brain temperature was maintained at mild hypothermia (33.0-34.0 °C) until 60 h after surgery. Once ice-water mattress was withdrawn, the brain temperature of PH group exhibited a significant increase compared with sham group at 72 hours. Nevertheless, the brain temperature of 8-OH-DPAT treated animals presented a similar but smoother reduced BT trend during the administration period. After drug withdrawal, the temperature of 8-OH-DPAT treated animals remained at a non-significant level compared with Sham group at 72 hours (Figure 2B).

### Therapeutic hypothermia improved the functional deficit caused by ICH

The motor deficiency of ICH rats was not ameliorated by physical or chemical hypothermia therapy in mNSS and rotarod test at first 24 hours following surgery. But at 72 hours, both the rats of CH and PH groups showed an improved mNSS score compared with ICH group. No significant difference was found in rotarod time between PH and ICH groups at 72 hours; whereas the 8-OH-DPAT treatment significantly alleviated the reduced rotarod time of ICH rats (Figure 2D and 2E).

In Morris water maze test, the escape latency was employed to assess learning and memory deficits in ICH animals. During training period, the escape latency of ICH group was significantly extended compared with Sham group. At days 17 and 18, the rats of CH group exhibited significantly shorter escape latency than ICH animals. For PH-treated animals, the therapeutic benefit on ICH rats was only identified on the last day of training period (Day 18) (Figure 2C).

### Morphological lesion induced by ICH was alleviated by hypothermia therapy

**Figure 3:**
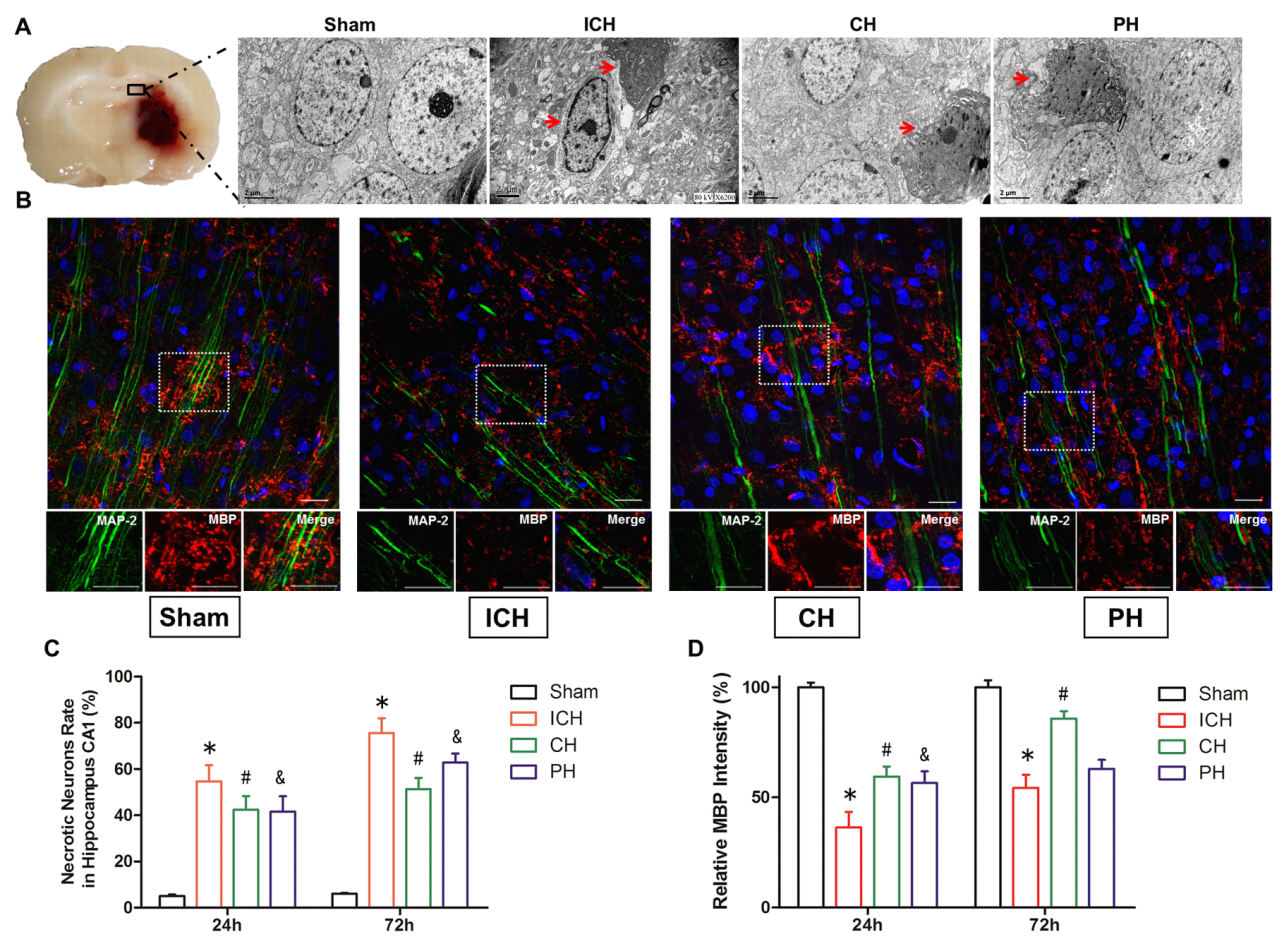
Neuronal necrosis and demyelinating injury induced by intracerebral hemorrhage (ICH) was improved by hypothermia **A.** Electron microscopic visualization of the typical neuronal necrosis in the hippocampal CA1 region of four groups at 72 h after onset (indicated by red arrows, Scale bar = 2 µm.). **B.** Immunohistochemical visualization of myelin basic protein -positive (MBP^+^) myelinating clusters at 72 h after onset (Scale bar = 100 µm). **C.** Quantitative analyses of identified necrotic hippocampal neurons at 24 and 72 h after surgery. **D.** Quantitative analyses of the proportion of myelinated axons. * *p* < 0.05 ICH group *vs*. Sham group. # *p* < 0.05 CH group *vs*. ICH group. &*p* < 0.05 PH group *vs*. ICH group. One-way ANOVA and Student-Newman-Keuls analysis. *n* = 7 per group at the corresponding time points.

As determined with an electron microscope, a substantial extent of ultrastructural damage was identified in the hippocampal CA1 area of ICH animals at 24 and 72 hours after surgery. The proportion of necrotic neurons intensely increased at 72 hours following ICH. Both the chemical and physical hypothermia decreased the number of necrotic neurons in the hippocampal CA1 area at early stage of ICH (Figure 3A and 3C).

The results of Immunofluorescence showed intracerebral hematoma led to a demyelinating lesion (lower intensity of MBP) on the projecting axons *via* hematoma at 24 and 72 hours after ICH (Figure 3B). At 24 hours, CH and PH therapies both improved the demyelinating cluster compared with ICH group. However, physical hypothermia had no benefit on the expanded white matter injury at 72 hours; at the same time, the demyelination lesion was significantly reduced by 8-OH-DPAT treatment (Figure 3D).

### Hypothermia therapy preserved the BBB breakage and suppressed the proinflammatory cytokine expression

**Figure 4:**
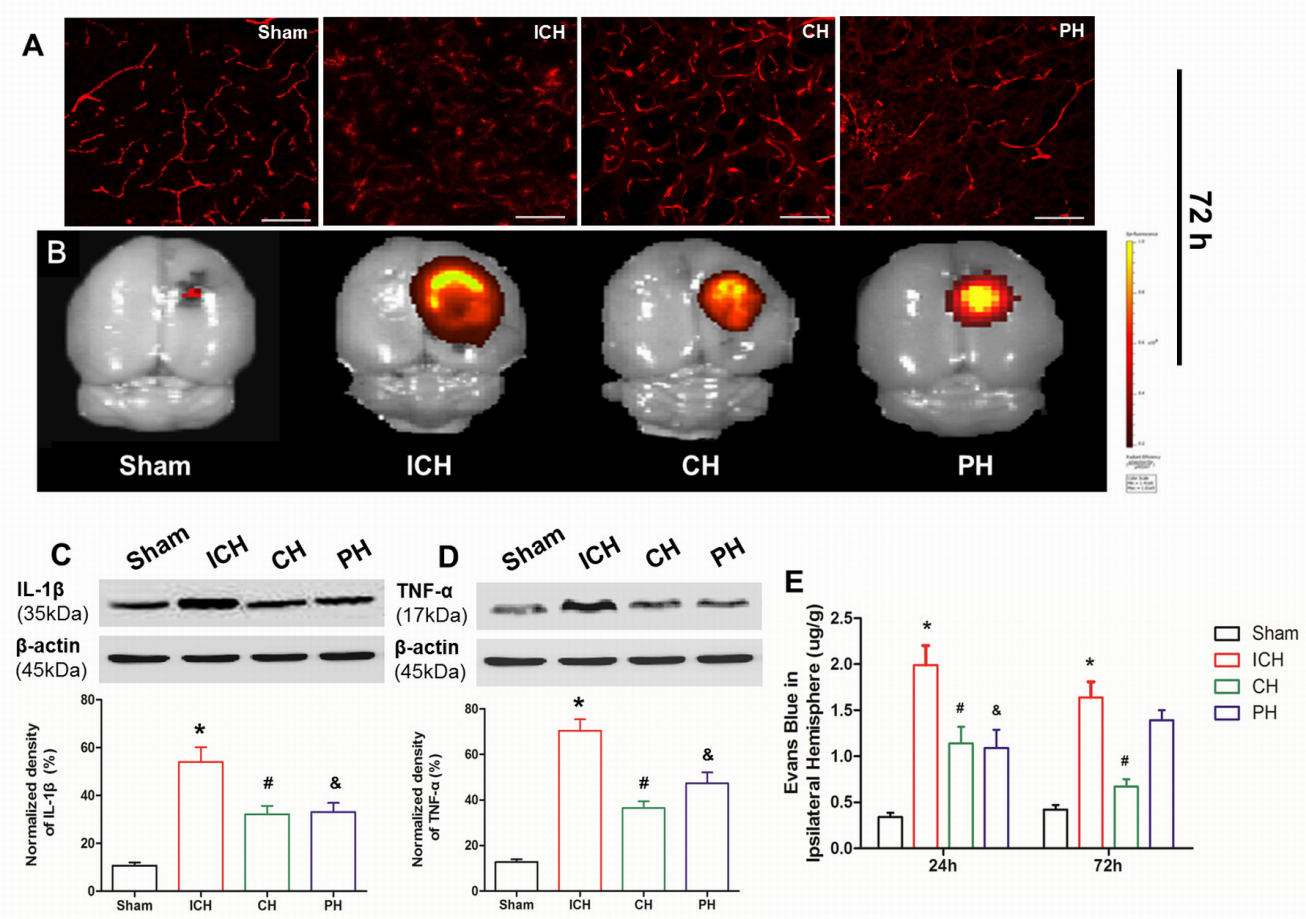
Effects of hypothermia on early blood-brain barrier damage following intracerebral hemorrhage (ICH) **A.** Immunohistochemical visualization of leaked Evans Blue (EB) outside the capillary vessels at 72 h after infusion. **B.** EB content in the whole brain was imaged using an IVIS system. Protein expression of **C.** IL-1β and **D.** TNF-α in para-hematoma brain tissue detected *via* western blotting at 72 h after ICH. **E.** Quantitative analyses of EB content in the ipsilateral hemisphere at 24 and 72 h. * *p* < 0.05 ICH group *vs*. Sham group. # *p* < 0.05 CH group *vs*. ICH group. &*p* < 0.05 PH group *vs*. ICH group. One-way ANOVA and Student-Newman-Keuls analysis. *n* = 7 per group at the corresponding time points. Scale bar = 2 µm.

The ICH-induced BBB breakage was investigated using Evans blue staining and western-blot. The quantitative data demonstrated that the EB leakage of the ipsilateral hemisphere was acutely increased at 24 and 72 hours post-ICH induction (Figure 4E). As shown in the immunofluorescence and IVIS Spectrum imaging, the EB dye permeability was substantially leaked to extra-capillary wall and focused around the capillary vessels near the hematoma at 72 hours post-ICH induction. However, the extravascular EB leakage observed by immunofluorescence was reduced both of CH and PH treatment (Figure 4A), and the volume of residual EB dye was decreased compared with ICH rats through IVIS Spectrum imaging (Figure 4B). The quantitative analysis indicated that chemical and physical hypothermia both reduced EB leakage at 24 hours following ICH. But EB permeability of PH group abnormally increased at 72 hours; while EB leakage of 8-OH-DPAT treated animals was still at low level (Figure 4E). Because the inflammatory response after ICH played a key role in the early BBB breakage, proinflammatory mediators, including IL-1β and TNF-α, were examined *via* western blot at 72 hours. Both chemical and physical hypothermia significantly reduced the increased expression of these proinflammatory cytokines in the para-hematoma tissue (Figure 4C and 4D).

### 8-OH-DPAT increased the firing rate of warm-sensitive neurons specifically *via* 5-HT1a receptor activation at PO/AH area

**Figure 5:**
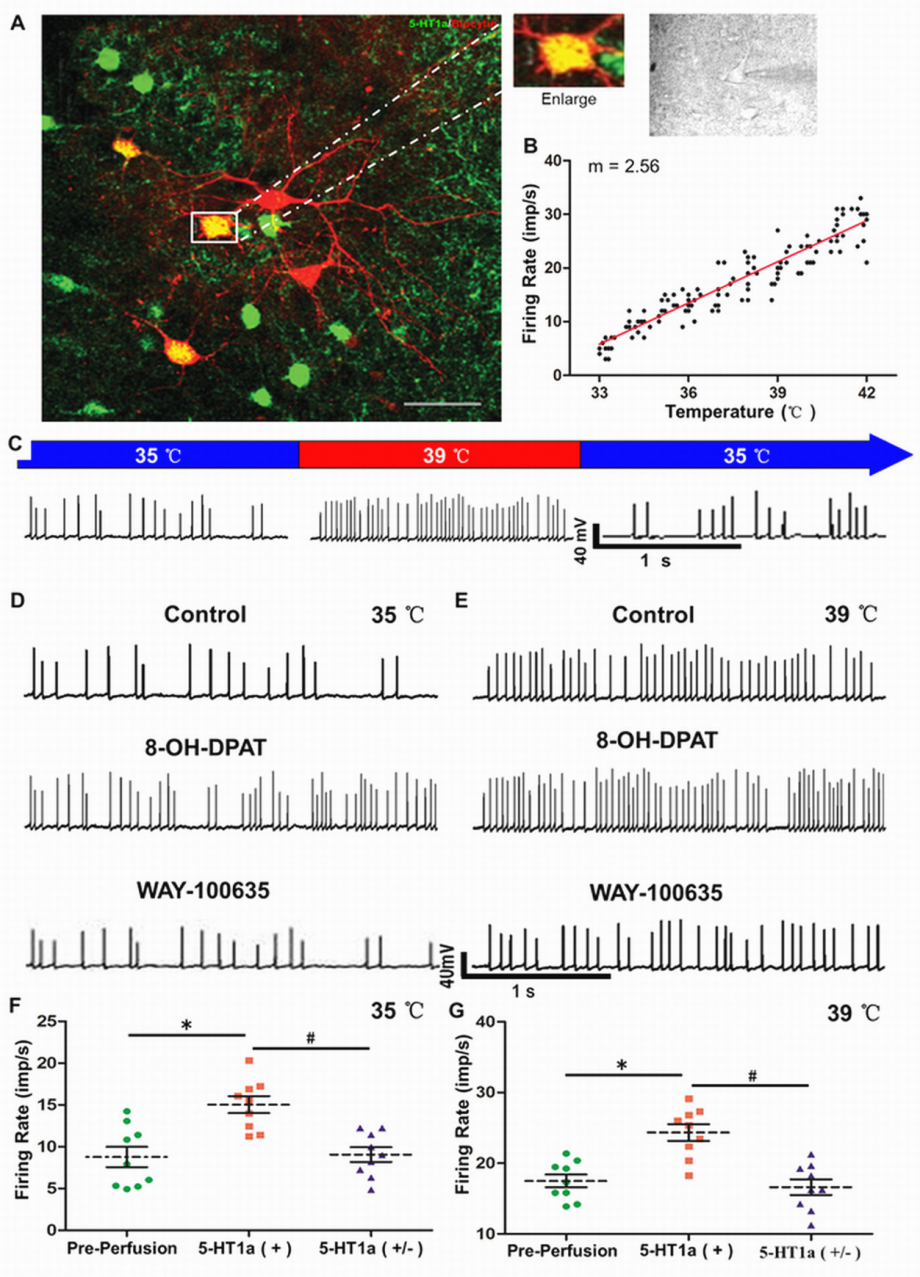
Roles of 5-HT1a receptor in spontaneous firing of warm-sensitive neurons in PO/AH area **A.**PO/AH neurons labeled by biocytin were visualized *via* immunofluorescence. **B.**Spontaneous firing of warm-sensitive neurons varying with temperature was recorded as a scatter plot (*n* = 17). **C.** Representative changes in warming-sensitive neuron with temperature. Perfusion of 8-OH-DPAT (1 μm) induced an increased firing frequency of warm-sensitive neurons, which was inhibited by WAY-100635 (1 μm) at 35 **D.** and 39 °C **E.** Quantitative analyses of firing rates of warm-sensitive neurons with 5-HT1a activation and inhibition at 35 **F.** and 39 °C **G.**. * *p* < 0.05 Pre-perfusion group *vs*. 5-HT1a (+) group. # *p* < 0.05 5-HT1a (+) group *vs*. 5-HT1a (-) group. Paired *t*-test. *n* = 9 per group. Scale bar = 2 µm.

The spontaneous firing rate changes with the rapid, periodic, temperature cycle were recorded in 73 neurons of the PO/AH area. The warm-sensitive neurons comprised 23.29 % (17) of the recorded neurons (Figure 5B and 5C). Using laser confocal scanning, approximately 22 % the 5-HT1a positive cells merged with the florescent signal of biocytin (Figure 5A). Nine confirmed warm-sensitive neurons were successively perfused with 1μm 8-OH-DPAT and WAY-100635 (a specific 5-HT1a antagonist). The spontaneous firing rates were respectively recorded at 35 and 39 °C (Figure 5D and 5E). After perfusion with 8-OH-DPAT, the firing rate was significantly decreased at different temperatures. Blocking 5-HT1a receptor with WAY-100635, the firing rate of warm-sensitive neurons was substantially decreased (Figure 5F and 5G).

## DISCUSSION

The current findings demonstrated that early increase in brain temperature was correlated with worse neurological outcomes in ICH patients. The animal study demonstrated that PH or CH therapy both reduced the increased temperature during the period of treatment. However, a rebound effect was identified during rewarming of physical hypothermia, while the brain temperature of CH group remained smooth following drug withdrawal. 8-OH-DPAT exhibited an advantage compared with PH therapy in neurological impairment tests, such as the rotarod test and Morris water maze. Additionally, fewer fractured axons and less BBB damage were identified in CH group post-ICH induction, whereas PH therapy failed to improve those early brain injuries at 72 hours. Furthermore, the warm-sensitive neurons in PO/AH area were verified the presence of 5-HT1a receptors and 5-HT1a activation specifically increased its spontaneous firing rate. According to these results, 8-OH-DPAT specifically activated the warm-sensitive neurons and acted as an endogenous cooling agent to act a gentle hypothermia neuroprotection post-ICH induction without post-withdrawal side-effects. The safety of therapeutic hypothermia was also confirmed by the physiological parameters of experimental animals before and after therapy (Table 2).

**Table 2:** Physiological parameters of experimental animal before and after surgery: No significant difference was observed between each group during therapy

	Before (*n* = 6)	CH-3d (*n* = 6)	PH-3d (*n* = 6)
Weight (g)	289.6 (17.8)	285.6 (15.4)	283.2 (16.7)
MABP (mm Hg)	101.4 (14.0)	109.0 (15.8)	107.1 (20.0)
pH	7.42 (0.06)	7.44 (0.03)	7.40 (0.03)
PaO2 (mm Hg)	95.9 (8.4)	93.9 (5.4)	96.6 (4.2)
PaCO2 (mm Hg)	40.7 (5.8)	38.4 (5.1)	40.6 (4.0)
Glucose (mM)	6.8 (0.6)	7.2 (0.6)	6.9 (0.9)
WBC count×10^3^/mmc	8.2 (0.5)	8.0 (1.0)	8.2 (1.5)
RBC count ×10^6^/mmc	6.2 (1.1)	6.4 (1.2)	6.0 (1.6)
PLT count ×10^3^/mmc	111.9 (11.4)	109.7 (10.6)	103.8 (10.8)

Values are presented as proportions, mean (SD), or median (quartiles). MABP: Mean Arterial Blood Pressure; WBC: white blood cell; RBC: red blood cell; PLT: platelet; mmc: cubic millimeter.

As is recently reported by the American Heart Association Stroke Council: hyperthermia ( > 37.6 °C) occurred in approximately one-third of acute stroke patients within the first 24 h and resulted in a 2-fold increase in the short-term mortality and worse neurological deficits [27]. Temperature modulation is recommended as a necessary measure to control increased ICP caused by intracerebral hemorrhage in NCU [28]. Nearly all electrical activities in CNS are susceptible to increased temperature, which would cause a 26-fold increase in BBB permeability and irreversible damage of cultured neurons [29]. The hyperthermia following brain injury was also noted as a detrimental factor to para-lesion edema formation and inflammatory cell infiltrates [30].

Multiple factors contributed to the increased brain temperature at early stage of ICH. As the venous system is more susceptible to increased ICP, The heat produced by nervous activity was intracranially accumulated because of an impaired heat outflow by the venous system post-ICH induction [31]. The up-regulation of mitochondrial uncoupling proteins (UCPs) secondary to ICH resulted in dysfunctions of the oxidative phosphorylation; thus, the electric potential energy of electron transfer was transformed into thermal energy instead of ATP [32]. Moreover, the neuro-inflammation was tightly linked to increased local temperature, which indicates that early cooling measures represent a promising treatment for early brain injury [33]. However, when therapeutic hypothermia was transformed from bench to bedside, no difference or even higher mortality rates were identified in hypothermia-treated group in recent randomized trials [10, 11].

*Via* the regulation of neuro-endocrine system, the thermoregulatory set-point could be subsequently pharmacologically reset by the heat-regulating neurons in PO/AH area [34]. The putative role of thermo-transient receptor potential (TRP) family in hypothalamic neuronal thermosensitivity has been suggested, which indicated TRPV1 immunoreactivity in PO/AH area [35]. Rinvanil, a synthetic TRPV1 agonist, demonstrated an effect of hypothermia and permanent neuroprotection in stroke animal model [36]. However, the repeated doses with a high frequency are required during treatment and excessive TRPV1 activation has been reported to trigger apoptotic cortical neuron death and reactive oxygen species formation, which limited its clinical applications [37]. The second-generation neurotensin receptor (NTR) agonist has been demonstrated with a therapeutical effect of pharmacological induced hypothermia on different animal models of CNS injuries in a broad therapeutic window, indicating that it is endowed with promising potential for clinical translational research [16-18].

The neuroprotective action of 5-HT1a agonists against hemorrhagic stroke has been observed before, but the underlying mechanism of its neuroprotection remained unknown. BAY X3702, a high affinity 5-HT1A agonist, was reported to alleviate the focal ischemic brain damage caused by acute subdural hematoma in rats [38]. Olsen AS *et al.* have shown that buspirone (5-HT1a agonists) has a narrow therapeutic dose response for improving cognitive and histological deficits induced by traumatic brain injury [39]. The present studies indicated that 5-HT1 receptor was expressed at warm-sensitive neurons in PO/AH area and participated in regulation of heat-regulating neurons (Figure 5). Targeting this physiological accommodation of body temperature, 8-OH-DPAT didn’t cause a vigorous thermoregulatory defense which led to the major side-effects of PH, including shivering [13], insulin resistance [40] and electrolyte disturbances [41]. The drastic brain temperature alteration during the rewarming was avoided in 8-OH-DPAT-induced hypothermia, which also avoided the rebound of ICP (Figure 2). The gentle rewarming process may be due to the smoothly varying characteristics of hypothalamic 8-OH-DPAT concentration after withdrawal, which needs further investigation. The cooling agents that target 5-HT system, which have been clinically proven with wide safety scopes by SSRIs, could make a promising early treatment for acute cerebrovascular diseases, especially in first-aid. However, the induction of cooling by 8-OH-DPAT treatment was relatively slow (nearly 12 hours according Figure 2), which may influence its effect on acute ischemic stroke.

In summary, our study indicated that chemical hypothermia induced by 8-OH-DPAT have a superior neuroprotection compared with physical cooling in rat ICH model. With a similar pharmacological action of SSRIs, chemical hypothermia that targets the 5-HT1a in PO/AH area may represent a promising therapeutic approach with minimal side-effects for ICH patients.

## MATERIALS AND METHODS

### Clinical study

To evaluate the effect of early increased brain temperature (BT) on hematoma absorption, para-hematoma edema (PHE) and National Institutes of Health Stroke Scale (NIHSS) score, twenty-six ICH patients were enrolled and randomized into normal BT and increased BT groups in a double blind method between September 2015 and January 2016. The temperature data were collected at a fixed point which is 1 cm lateral to outer canthus at ipsilateral tempus on admission and 7 days after ICH. A near-infrared spectrum (NIS) thermography detector (Fluke VT04, Fluke Co., Ltd., Fred, WA, US) which allows a rapid and non-contact recording of the irradiated energy released from body was applied to measure the head temperature [42]. Thus, an increased BT was defined as more than 37 °C, since the collected temperature on the surface of head is lower than the core temperature. The rectal temperature of enrolled cases was routinely monitored. The hematoma was visualized using a 1.5T MRI scanner (Magnetom Trio, Siemens, Germany) on the day of admission and 7 d after ICH. The relative PHE was defined as PHE volume divided by hematoma volume. The protocols and procedures were approved by the Institutional Ethics Committee of Southwest Hospital, and informed consent was obtained from all subjects or authorized caregivers. The patients with signs of infection, such as raised body temperature and increased leukocyte, were excluded.

### Inclusion and exclusion criteria

The inclusion criteria were as follows: 1) the brain temperature was collected within the first 24 h of stroke onset; 2) the diagnosis was confirmed by cranial MRI; and 3) the hematoma volume in the basal ganglia was between 20 and 40 ml. The exclusion criteria were as follows: 1) the patients were < 18 y or > 80 y; 2) signs of infectious fever; 3) a craniotomy was chosen; 4) the patient was in a coma or died during follow-up; 5) ICH was caused by a brain tumor, trauma, drug abuse, coagulation abnormalities, anticoagulation therapy, or vascular malformations; or 6) the study protocol was violated by the participator. At admission to the hospital, the surface temperature of the ipsilateral tempus was surveyed to screen for an increased BT.

### Rat model of ICH

One hundred and eighty-nine adult male Sprague-Dawley (SD) rats (250-320 g) and twenty-one infant SD rats (P21-P24) were used with a completely randomized, double blind controlled method in current experiments. The animal experiment procedures were in compliance with the China Animal Welfare Legislation and were approved by the Third Military Medical University Committee on Ethics in the Care and Use of Laboratory Animals. ICH rat model was established by autologous blood injection into basal ganglia. Briefly, after anesthetizing with a pentobarbital (50 mg/kg), a 29-gauge needle was inserted into the right basal ganglia (coordinates: 0.2 mm anterior, 5.5 mm ventral, and 3.5 mm lateral to midline) with head was mounted on a stereotaxic frame; 100 µl autologous arterial blood were infused at a rate of (5 µl/min) with a micro-infusion pump. The rats in sham group only received a needle injection in the same location as ICH group. All surgical procedures were conducted under aseptic conditions.

### Hypothermia protocol

Before surgery, the experimental animals were randomly grouped as follows: sham-operated (Sham), vehicle-treated ICH rats (ICH), 8-OH-DPAT treated ICH rats (CH), and physical hypothermia treated ICH rats (PH). 8-OH-DPAT (Sigma-Aldrich Co., St. Louis, MO, US.) was dissolved in sterile saline and administered i.p. 10 mg/kg every 12 h for 48 hours according to previous study [43]. First dose was administered at 2 hours after surgery. The equal dose of sterile saline was i.p. administered in Sham and ICH groups at same intervals. Physical hypothermia was initiated without sedation at 2 hours after surgery, and the body temperature was maintained at 33.0-34.0 °C for 48 hours. ICH animals were slowly induced to reach target rectal temperature within 12 hours using an ice-water mattress, and the rewarming process required 12 hours after 48 hours of mild hypothermia.

### Behavioral function examination

The motor deficiency caused by ICH was assessed using a modified Neurological Severity Score (mNSS) and rotarod test at 24 and 72 hours after surgery. The mNSS test score is based on a series of motor, sensory, reflex, and balance tests. The valuation of the motor function deficiency was graded based on a score of 0 (minimal deficit) to 18 (maximal deficit). The rats that participated in rotarod test were trained for running on the rotarod (diameter, 10 cm; constant speed, 8 rpm) for 180 s prior to surgery. The rotarod test was conducted at 2 hours prior to surgery and 24 and 72 hours after surgery; the running time was recorded until falling (or 180 s). Five trials were performed for each rat, and the average of the top 3 latency times was divided by the latency prior to surgery.

The Morris water maze test was adopted to assess learning and spatial memory deficits at 14 to 18 days as previously described [44]. The platform (6 cm in diameter, 1 cm below the water surface) was submerged in the target quadrant of swimming pool (160 cm in diameter, 50 cm in height). Animals were placed in the swimming pool facing the wall in four different quadrants. All test animals were allowed to swim freely for 120 s to adapt to the water maze environment, which had a temperature maintained at 22 ± 2 °C. If rat climbed onto the hidden platform within 120 s, the escape latency was recorded, and rats were allowed to remain on the platform for 15 s. For rats that failed to find the platform within given time, the experimenter helped them to rest on the platform for 15 s, and the escape latency was recorded as 120 s. All behavioral tests were performed by two investigators blinded to animal grouping.

### Transmission electron microscopy

With deep anesthetization, the brain tissues of ipsilateral hippocampal CA1 area were collected after transcardial perfusion with PBS and 4% phosphate-buffered paraformaldehyde at 24 and 72 hour after ICH. The samples were dissected and post-fixed with 2% glutaraldehyde and 2% formaldehyde PBS overnight at 4°C. The dehydrated samples were subsequently impregnated with epoxy resin and sectioned for the target area under a light microscope. After double-staining with uranyl acetate and lead citrate, the samples were examined with an H7100 transmission electron microscope (Hitachi, Tokyo, Japan); the necrotic neurons were calculated as the average of eight visual fields per rat.

### Immunofluorescence

Following transcardial perfusion with PBS and 4% phosphate-buffered paraformaldehyde, the white matter tissues surrounding the hematoma were removed and fixed in 4% phosphate-buffered paraformaldehyde for 3 days; the samples were subsequently dehydrated by 20/30 % sucrose PBS for an additional 3 days. The samples were sectioned in 20 µm thick slices and were incubated in anti-microtubule-associated protein-2 (MAP-2) primary antibody (1:500; Santa Cruz, US) and anti-myelin basic protein (MBP) primary antibody (1:500; R&D System, US) at 4 °C overnight. The slices were probed with secondary antibody (Goat anti-mouse GFAP; Jack Immunoresearch) and DAPI after washing with PBS. The observation of projecting nerve fiber bundle *via* hematoma was conducted with a laser confocal microscope (LSM780, Zeiss, Germany) and analyzed semi-quantitatively with Image J. The regions of interest (ROI) were set in the white matter area around hematoma to measure the MBP fluorescence intensity. Average fluorescence intensity was computed from 3 randomly selected ROIs, and 3 consecutive sections were analyzed for each rat. The relative MBP intensity was calculated as (the fluorescence intensity of ICH group / the fluorescence intensity of Sham group) ×100%.

### Evans blue fluorescence imaging and extravasation

The BBB damage induced by ICH was evaluated *via* the extravasation of Evans blue (EB) dye. The EB dye (2%, 5 ml/kg; Sigma-Aldrich Co., St. Louis, MO, USA) was injected into the jugular vein at 2h prior to execution. Under anesthesia, The whole brains were dissected for coronal brain sections (20 um) using the same procedure as immunofluorescence. Excitation and emission filters for rhodamine fluorescence were applied to determine the auto-fluorescence of EB with a laser confocal microscope (LSM780, Zeiss, Germany).

For whole brain imaging, an *in vivo* imaging system (IVIS) (PerkinElmer, Inc.) was applied to determine the EB extravasation under the same excitation and emission filters used with the laser confocal microscope. In the quantitative analysis of EB extravasation, the brain samples were divided into ipsilateral and contralateral hemispheres for homogenate and weighed after transcardial perfusion with PBS. The samples were subsequently homogenized in dimethyl formamide (5 %) at 15,000 rpm for 30 min and incubated at 60 °C overnight. The collected supernatant was then spectrophotometrically quantified for the EB content at O.D. 620 nm (Thermo Scientific, US).

### Western blot analysis

For protein analysis of proinflammatory cytokines in the para-hematoma tissues, the animals was euthanized *via* intracardial perfusion of PBS and the brain tissues around hematoma were collected. Proteins obtained from six rats per group were extracted in a buffer (1% Triton X-100 with 1 mg/ml leupeptin, 1 mM PMSF, and 1 µg/ml pepstatin; pH 7.4) and centrifuged at 12,000 g for 30 min at 4 °C. Equivalent protein amounts (20 μg) were loaded in each lane of SDS-PAGE gels. Following gel electrophoresis, the protein ladders were transferred to a PVDF membrane (Millipore, US), blocked with 10% bovine serum albumin (BSA), and incubated in primary antibodies and the corresponding secondary antibodies. The following antibodies were used: anti-IL1β (1:1000; R&D System, US) and anti-TNF-α (1:1000; Cell Signaling technology, US). The immunoreactive bands were visualized with an ECL kit (Amersham Biosciences, Arlington Heights, IL) following the manufacturer’s instructions. The data were analyzed *via* densitometry with ImageJ software. β-actin was used as an internal loading control (1:1000; Santa Cruz, US).

### Whole-cell clamp recordings

The coronal brain slices, including the PO/AH area of the hypothalamus, were rapidly prepared from the anesthetized SD rats (P 10-15). The brain slices were cut into 300 µm thick slices using an oscillating tissue slicer (Leica, VT1000) and were transferred to a recording chamber that was continuously perfused with artificial cerebrospinal fluid (aCSF) prior to recording. The aCSF was composed of the following (in mM): 124 NaCl, 2.5 KCl, 26 NaHCO3, 1.25 K2HPO4, 2 MgCl2, 2 CaCl2, and 10 D-glucose, saturated with 95% O2 / 5% CO2 to pH 7.4 at room temperature. The neurons in the PO/AH area were targeted for clamping with the assistance of a microscope equipped with Leica infrared-differential interference contrast (IR-DIC) optics and a water-immersion objective. The patch pipette (3-7 MΩ) was filled with an internal solution that contained the following (in mM): 125 potassium gluconate, 20 KCl, 4 MgCl2, 0.5 CaCl2, 10 HEPES, 1 EGTA, and 5 D-glucose, pH 7.2-7.4. The identified warm-sensing neurons were intracellularly labeled with 0.5% biocytin (Invitrogen, US) in the pipette solution. The signal was amplified with an EPC10 amplifier (HEKA Elektronik, US) and analyzed with Igor Pro v.4.03 (WaveMetrics).

### Thermoelectricity record and dye-coupling

The thermosensitivity of PO/AH neurons was characterized by the spontaneous firing frequency as well as rapid, periodic temperature fluctuations (36.0-42.0 °C). As Tang et al. reported [45], the linear regression coefficient (thermal coefficient) or slope (m), which was determined by the firing rate varying with the temperature, was used to define the neuronal thermosensitivity. A warm-sensitive neuron was identified if the thermal coefficient was at least 0.8 imp/s/ °C. After the thermoelectrical property and control spontaneous firing activity were fixed, 1 μm 8-OH-DPAT (5-HT1a receptor agonist) and 1 μm WAY-100635 (5-HT1a receptor antagonist, Sigma-Aldrich Co., St. Louis, MO, USA) were successively added to the perfusing aCSF to investigate the effect of 5-HT1a on the thermoelectrical property of warm-sensitive neurons.

The slices with the identified warm-sensitive neurons intracellularly injected with biocytin were fixed in 4% phosphate-buffered paraformaldehyde at 4 °C for 2 hours. The slices were subsequently immersed in 2% Triton X-100, 2% BSA and 10% donkey serum dissolved in PBS at pH 7.4 for 2 hours. The slices were incubated in rabbit monoclonal antibody to 5-HT1a (1:500; Abcam, US) at 4 °C overnight. After washing, the slices were visualized using dylight-488 conjugated donkey anti-rabbit IgG (1:200; Jackson ImmunoResearch) and Cy3-conjugated streptavidin (1:200; Jackson ImmunoResearch) at 4 °C for 4 hours. The observation and images were acquired using confocal microscopy (LSM780, Zeiss, Germany) and were analyzed with Zeiss imaging software.

### Statistical analysis

All data are presented as mean ± standard deviation (SD) and analyzed with SPSS 17.0 software. Statistical analyses were performed by two-way analysis of variance (ANOVA) (time × treatment) followed by Bonferroni post hoc test with a significance cut off of α /n for multiple comparisons. The statistical significance was two-tailed, and differences were considered significant when *p* < 0.05.

### Ethical approval

All procedures performed in studies involving human participants were in accordance with the ethical standards of the Institutional Ethics Committee of Southwest Hospital and with the 1964 Helsinki declaration. All national and institutional guidelines for the care and use of animals were followed.

## SUPPLEMENTARY MATERIALS FIGURE


