# Supplementary material for: 5-HT1a activation in PO/AH area induces therapeutic hypothermia in a rat model of intracerebral hemorrhage
Source: Oncotarget. 2017 Aug 16;8(43):73613–26. doi: 10.18632/oncotarget.20280 (PMC5650286; doi:10.18632/oncotarget.20280)
Supplement: Supplementary file 1 [file oncotarget-08-73613-s001.pdf]

5-HT1a activation in PO/AH area induces therapeutic hypothermia in a rat model of intracerebral hemorrhage

SUPPLEMENTARY MATERIALS

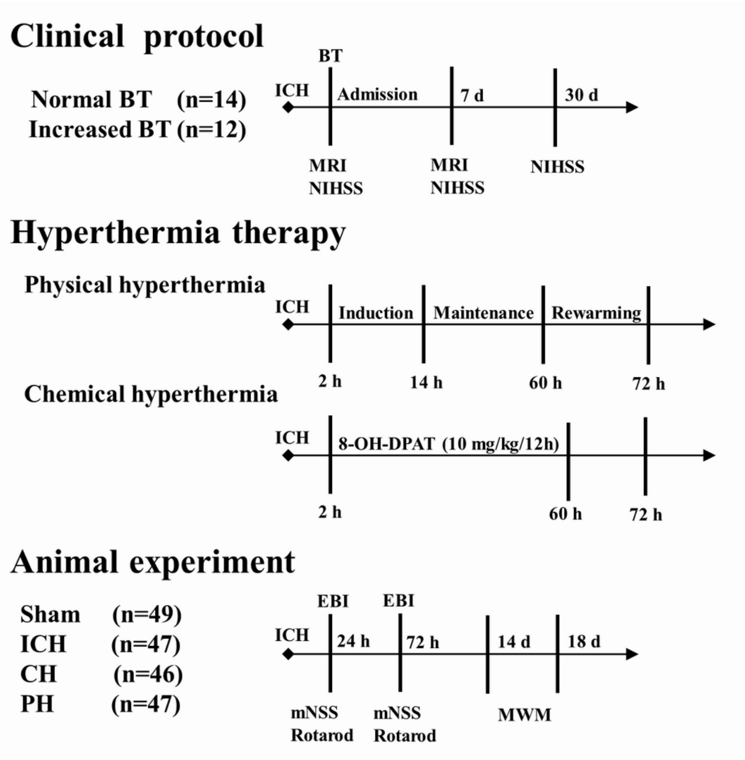

Supplementary Figure 1: Experimental protocol. BT: brain temperature; ICH: intracerebral hemorrhage; NIHSS: National Institutes of Health Stroke Scale; CH, chemical hypothermia; PH: physical hypothermia; EBI: early brain injury; mNSS: modified Neurological Severity Score; MWM: Morris water maze.
